# Development of a novel immune-related genes prognostic signature for osteosarcoma

**DOI:** 10.1038/s41598-020-75573-w

**Published:** 2020-10-27

**Authors:** Zuo-long Wu, Ya-jun Deng, Guang-zhi Zhang, En-hui Ren, Wen-hua Yuan, Qi-qi Xie

**Affiliations:** 1 Breast Disease Diagnosis and Treatment Center, Affiliated Hospital of Qinghai University & Affiliated Cancer Hospital of Qinghai University, No.29 Tongren Road, Xining, 810000 Qinghai China; 2 Guanghe Traditional Chinese and Western Medicine Hospital, Lanzhou, 730000 Gansu China; 3 Department of Orthopaedics, Second Hospital of Lanzhou University, Lanzhou, 730000 Gansu China

**Keywords:** Cancer, Computational biology and bioinformatics, Immunology, Molecular biology, Biomarkers

## Abstract

Immune-related genes (IRGs) are responsible for osteosarcoma (OS) initiation and development. We aimed to develop an optimal IRGs-based signature to assess of OS prognosis. Sample gene expression profiles and clinical information were downloaded from the Therapeutically Applicable Research to Generate Effective Treatments (TARGET) and Genotype-Tissue Expression (GTEx) databases. IRGs were obtained from the ImmPort database. R software was used to screen differentially expressed IRGs (DEIRGs) and functional correlation analysis. DEIRGs were analyzed by univariate Cox regression and iterative LASSO Cox regression analysis to develop an optimal prognostic signature, and the signature was further verified by independent cohort (GSE39055) and clinical correlation analysis. The analyses yielded 604 DEIRGs and 10 hub IRGs. A prognostic signature consisting of 13 IRGs was constructed, which strikingly correlated with OS overall survival and distant metastasis (p < 0.05, p < 0.01), and clinical subgroup showed that the signature’s prognostic ability was independent of clinicopathological factors. Univariate and multivariate Cox regression analyses also supported its prognostic value. In conclusion, we developed an IRGs signature that is a prognostic indicator in OS patients, and the signature might serve as potential prognostic indicator to identify outcome of OS and facilitate personalized management of the high-risk patients.

## Introduction

Osteosarcoma (OS) is a primary bone malignant tumor that most commonly affects children, adolescents, and young adults, and it also exhibits a predilection to occur in the metaphysis of long bones, and most commonly occurs in the distal femur (43%), proximal tibia (23%), or humerus (10%)^1^. Additionally, osteosarcoma is aggressive and often metastasizes to the lungs^2^. In the past 10 years, the incidence of OS has been annually increasing by 0.3%^3^, and it has been consistently ranked as the second deadliest cancer in adolescents and children^4^. Despite advances in multimodal therapy, the 5-year survival of osteosarcoma is approximately 60% to 70%, which has remained stagnant over the past three decades, patients with distant metastases still fare poorly, as the 5-year survival rate in these patients does not exceed 20%^5,6^. In addition, patients with the same clinical or pathological conditions receiving the same treatment regimen may have different clinical outcomes, due to their genetic heterogeneity^7^. Therefore, in-depth exploration of the molecular mechanisms behind the development of OS is crucial to finding effective prognostic biomarkers to guide patient risk stratification, which aligns with the concept advocated by precision medicine.

In recent years, biomolecules and risk models have been used to evaluate the prognosis of OS^8–11^. However, they have not yet been used in clinical practice because of unavoidable limitations, such as overfitting due to small samples^12^. In recent decades, increasing evidence has indicated that the immune response is actively involved in OS occurrence and progression^13^. Immune genes act as pivotal regulator of immune response^14,15^. They maintain the body's self-tolerance by strictly regulating the immune function and reducing the damage inflicted on the surrounding tissues^16^. However, OS cell may use these immune genes to escape the immune system and achieve a favorable environment for their growth^13,17^. Given the critical role of immune molecules in OS prognosis, these immune-related genes (IRGs) deserve further study.

Here, were identified differentially expressed IRGs (DEIRGs) in OS and normal muscle tissue samples. Subsequently, an IRGs signature that can predict outcome of OS was constructed by using univariate Cox regression and iterative LASSO Cox regression analysis of DEIRGs. In addition, based on an independent cohort, the accuracy of IRGs signature in predicting the prognosis of OS patients was verified. Finally, we also evaluated the independence, repeatability, and clinical value of the IRGs signature in different clinical subgroups. Our results reveal the prognostic value of IRGs signature and provide promising prognostic indicator for OS.

## Results

### GO and KEGG pathways enrichment analysis of DEIRGs

**Figure 1:**
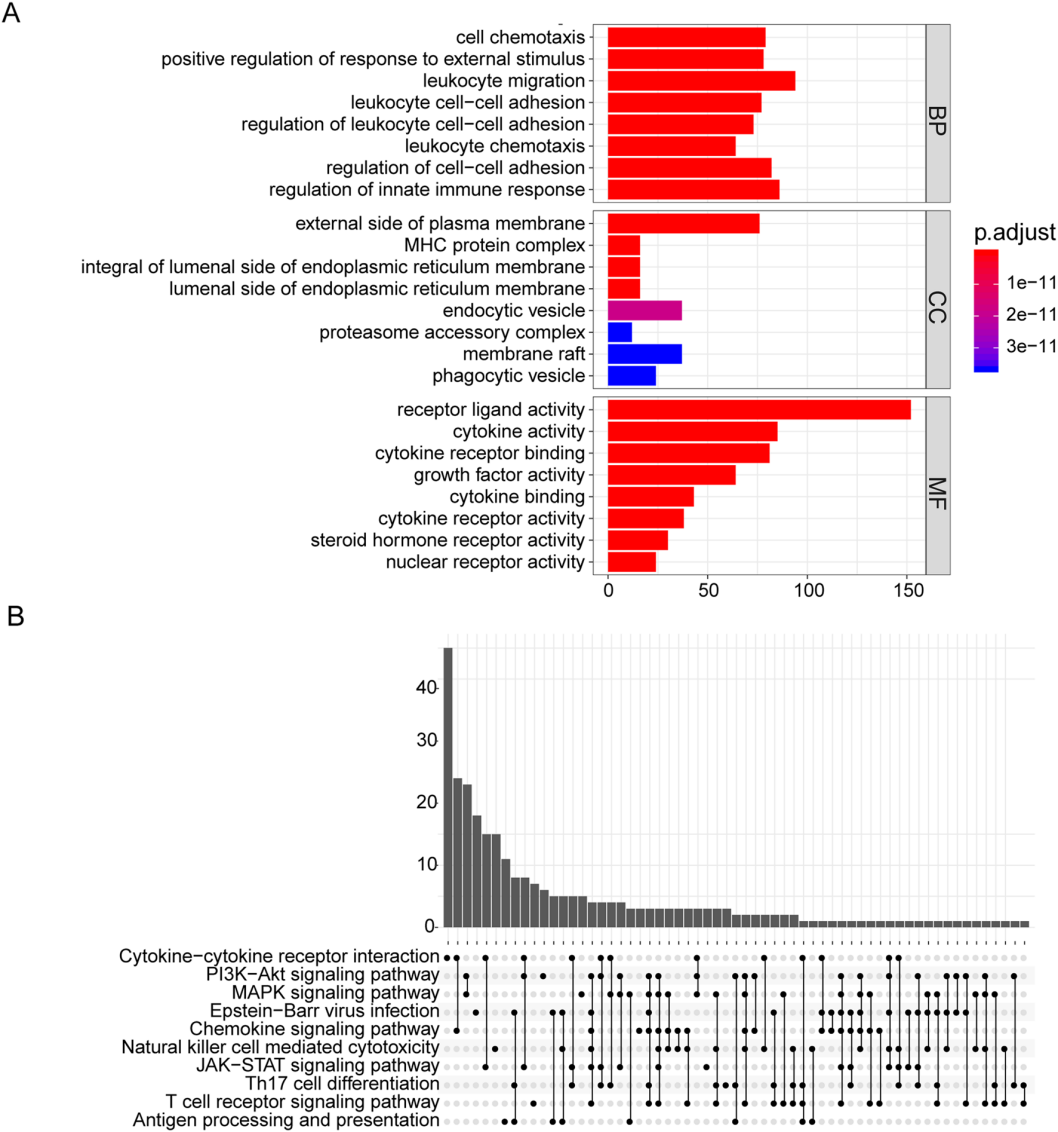
GO and KEGG analysis of DEIRGs. (**A**) GO enrichment analysis of DEIRGs. The color of the bar indicates p.adjust: the redder the color, the smaller the p.adjust value; the bluer the color, the larger the p.adjust value. The horizontal axis represents the number of DEIRGs under the GO term. (**B**) KEGG pathways enrichment analysis of DEIRGs. Significant gene (p.adjust < 0.05) enrichment to the 10 most important paths. p.adjust: adjusted P-value; BP: biological process; CC: cellular component; MF: molecular function.

All 604 DEIRGs were screened. GO analysis results showed that DEIRGs are involved in biological functions such as cell chemotaxis, leukocyte adhesion, and innate immune regulation. They were also determined to participate in cellular components such as the external side of the plasma membrane, MHC protein complexes, endoplasmic reticulum membranes, and phagocytic vesicles. Additionally, they are also found to be involved in molecular functions including receptor-ligand, cytokine, steroid receptor, and nuclear receptor activity (Fig. 1A). The KEGG analysis indicated that DEIRGs were mainly enriched in the following signaling pathways: chemokines, PI3K/AKT, MAPK, JAK-STAT, and natural killer (NK) cell-mediated cytotoxicity signaling (Fig. 1B).

### PPI network construction, hub IRGs screening, and functional similarity analysis of DEIRGs

**Figure 2:**
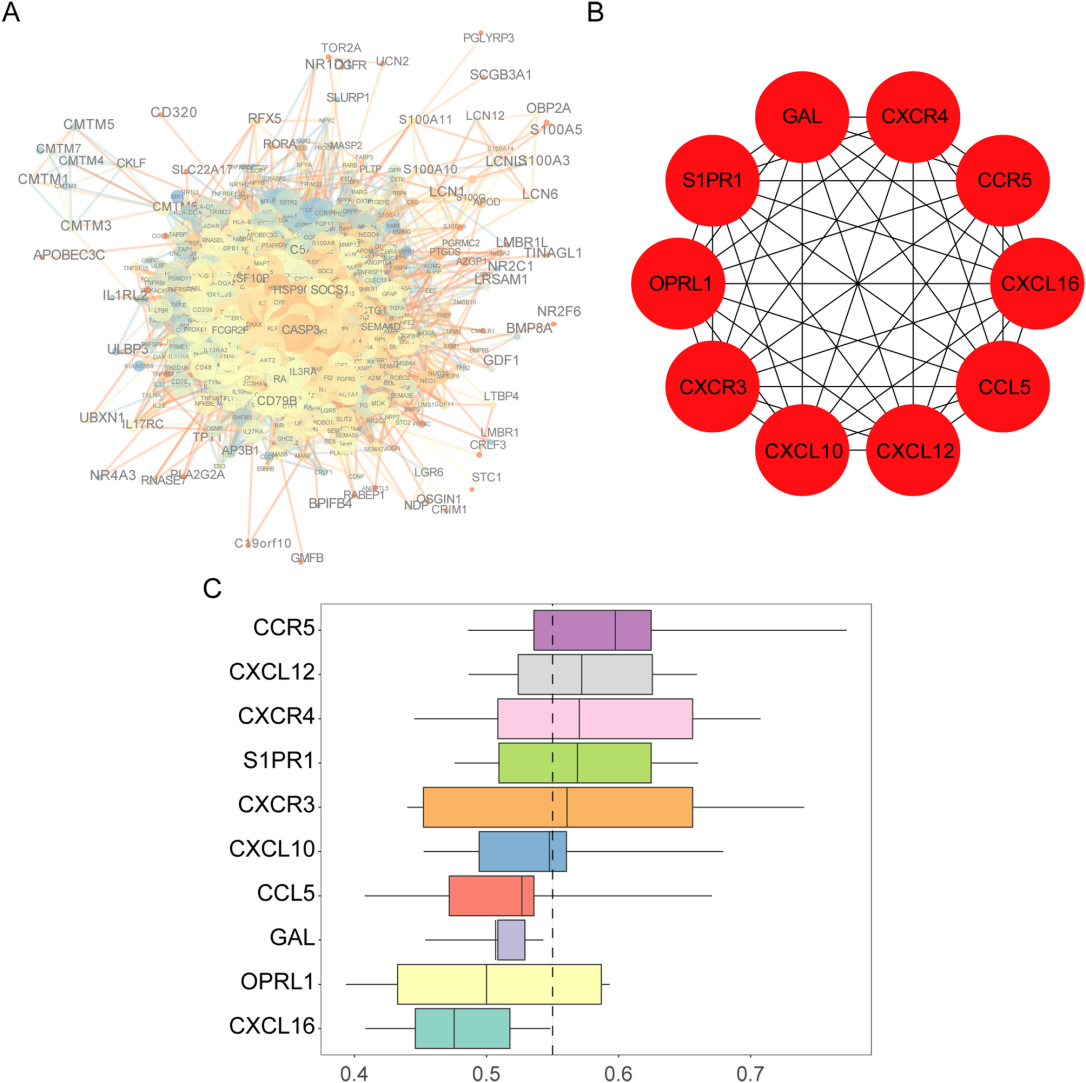
Protein–protein interaction (PPI), hub IRGs, and functional similarity analysis of DEIRGs. (**A**) PPI network. The size of a node represents the clustering coefficient, the color indicates the degree, the width of the line indicates the score; the color of the line represents co-expression. (**B**) Hub IRGs. The hub IRGs were the top 10 DEIRGs scored by the maximum correlation coefficient. (**C**) Functional similarities of 10 hub IRGs. The boxes indicate the middle 50% of the similarities; the upper and lower boundaries represent the 75th and 25th percentiles. The two ends of the line represent the maximum and minimum values. The dashed line represents the cut-off value of similarity.

The result of these analyses were shown in Fig. 2A. CASP3, TNFRSF10B, and HSP90 had a larger weight and a stronger correlation in the PPI network. Ten hub IRGs were obtained, namely CXCR4, CCR5, CXCL16, CCL5, CXCL12, CXCL10, CXCR3, OPRL1, S1PR1, and GAL (Fig. 2B, Table 1). To further recognize the closeness of the interactions between hub IRGs, which were ranked according to average functional similarity, as indicated by the results, CCR5, CXCL12, CXCR4, SIPR1, and CXCR4 were found to be hub genes with cut-off values greater than 0.55, and CCR5, CXCL12, and CXCR4 were the most closely related genes (Fig. 2C).

**Table 1:** Functions of 10 hub IRGs.

No	Symbol	Full name	Function
1	CXCL16	C-X-C motif chemokine ligand 16	CXCL16 is highly expressed in osteosarcoma tissues
2	CCL5	C–C motif chemokine ligand 5	High CCL5 expression is associated with osteosarcoma metastasis and poor prognosis of patients with osteosarcoma
3	CCR5	C–C motif chemokine receptor 5	CCR5 controls the proliferation or differentiation of osteosarcoma
4	GAL	Galanin and GMAP prepropeptide	The overexpression of Gal-1 is well established in many types of cancer progression like osteosarcoma, breast, lung, prostate, melanoma, etc
5	S1PR1	Sphingosine-1-phosphate receptor 1	Downregulated S1PR1 suppresses osteosarcoma metastasis and proliferation
6	CXCR4	C-X-C motif chemokine receptor 4	CXCR4-mediated osteosarcoma growth and pulmonary metastasis
7	CXCL12	C-X-C motif chemokine ligand 12	CXCL12 plays a critical role in mediating tumor progression and the immune response in osteosarcoma
8	CXCR3	C-X-C motif chemokine receptor 3	CXCR3 correlates with immune infiltration and predicts poor survival in osteosarcoma
9	CXCL10	C-X-C motif chemokine ligand 10	CXCL10 plays an important role in cancer and autoimmunity
10	OPRL1	Opioid-related nociceptin receptor 1	OPRL1 plays a key role of pain and injury perception

### Identification and assessment of the prognostic signature

**Figure 3:**
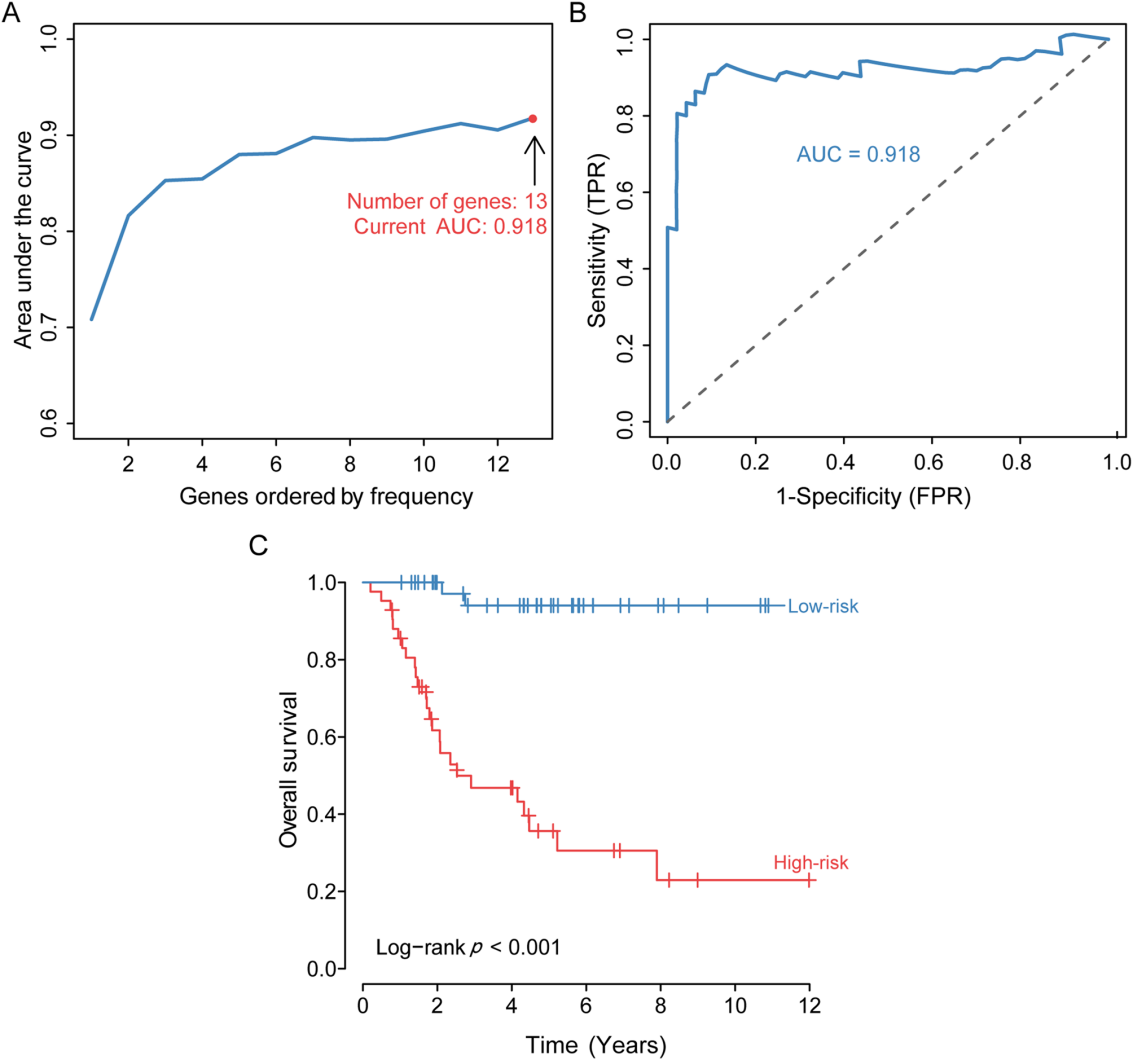
Development and assessment of the prognostic signature. (**A**) Construction of the prognosis-associated IRGs signature. The horizontal axis represents the gene frequency and the vertical axis represents the AUC. (**B**) Time-dependent ROC curve for prognosis-associated DEIRGs signature. The horizontal axis indicates the FDR, and the vertical axis indicates the TPR. (**C**) Kaplan–Meier survival curves of overall survival from the high-risk and low-risk groups. The horizontal axis represents survival time (y), and the vertical axis represents the survival rate (%). ROC: relative operating characteristic curve; AUC: area under the curve. FDR: false positive rate; TPR: true positive rate.

**Figure 4:**
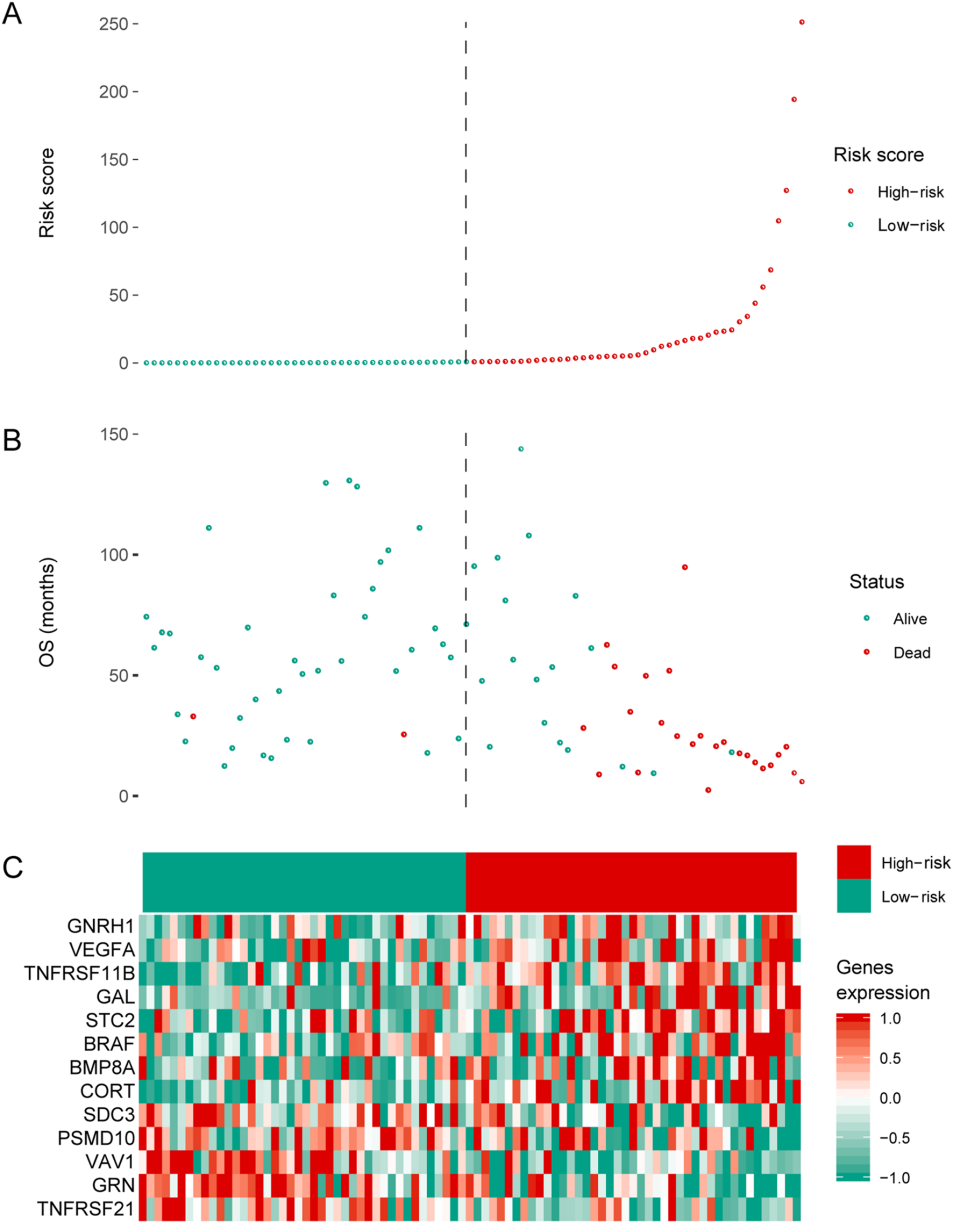
Prognostic analyses of high-risk and low-risk patients. (**A**) Risk score distribution of patients in the prognosis-associated IRGs signature. (**B**) Survival status scatter plots for patients in the prognosis-associated IRG signature. (**C**) Expression patterns of risk genes in the prognosis-associated IRG signature. Red means high expression, green means low expression. OS: overall survival.

To identify the optimal prognostic signature of OS based on IRGs, 82 prognostic-associated IRGs were identified by univariate Cox regression analysis of DEIRGs. Further, we identified the optimal prognostic signature that consisted of 13 prognosis-associated IRGs via the iterative LASSO Cox regression analysis (Fig. 3A, Table 2). ROC curve results showed that the accuracy of this signature in diagnosing OS prognosis was high (Fig. 3B, AUC = 0.918). The Kaplan–Meier curve indicated that the overall survival of patients in the high-risk group was markedly worse than that in the low-risk group (Fig. 3C, p < 0.001). According to the optimal signature, we obtained the risk score distribution (Fig. 4A), the survival status (Fig. 4B), and the expression characteristics of the immune genes of OS (Fig. 4C). Compared to the low-risk group, the high-risk group had more deaths. In addition, the expression levels of GNRH1, VEGFA, TNFRSF11B, GAL, STC2, BRAF, BMP8A, and CORT were higher in the high-risk group, whereas patients in the low-risk group expressed higher levels of PSMD10, TNFRSF21, GRN, VAV1, and SDC3.

**Table 2:** IRGs function in the prognostic signature.

No	Symbol	Full name	Function
1	BRAF	B-Raf proto-oncogene, serine/threonine kinase	Associated with progression and poor prognosis of several cancers
2	CORT	Cortistatin	Biological activities of anti-inflammation, antioxidation, antitumor activity
3	GAL	Galanin and GMAP prepropeptide	High expression is linked with the initiation and progression of prostate cancer and colorectal cancer
4	GRN	Granulin precursor	Inhibit the growth of hepatocellular carcinoma
5	STC2	Stanniocalcin 2	High expression is associated with progression and poor outcome of colorectal cancer
6	TNFRSF11B	TNF receptor superfamily member 11b	High expression is linked with a worse outcome of colorectal cancer
7	BMP8A	Bone morphogenetic protein 8a	High expression is associated with progression and poor survival of thyroid cancer
8	PSMD10	Proteasome 26S subunit, non-ATPase 10	Abnormal expression is linked with metastasis and worse survival of osteosarcoma
9	VEGFA	Vascular endothelial growth factor A	Promotes cell proliferation and migration, inhibits apoptosis of osteosarcoma
10	VAV1	Vav guanine nucleotide exchange factor 1	High expression is associated with the prognosis of invasive breast cancer
11	GNRH1	Gonadotropin-releasing hormone 1	Associated with vascular invasion and metastasis of osteosarcoma
12	SDC3	Syndecan 3	Overexpression inhibits the proliferation of mesenchymal tumor cells
13	TNFRSF21	TNF receptor superfamily member 21	Abnormal expression is coupled with growth, migration and invasion of osteosarcoma

### Comparison of the IRGs signature with other known prognostic biomarkers and verification in independent cohort

**Figure 5:**
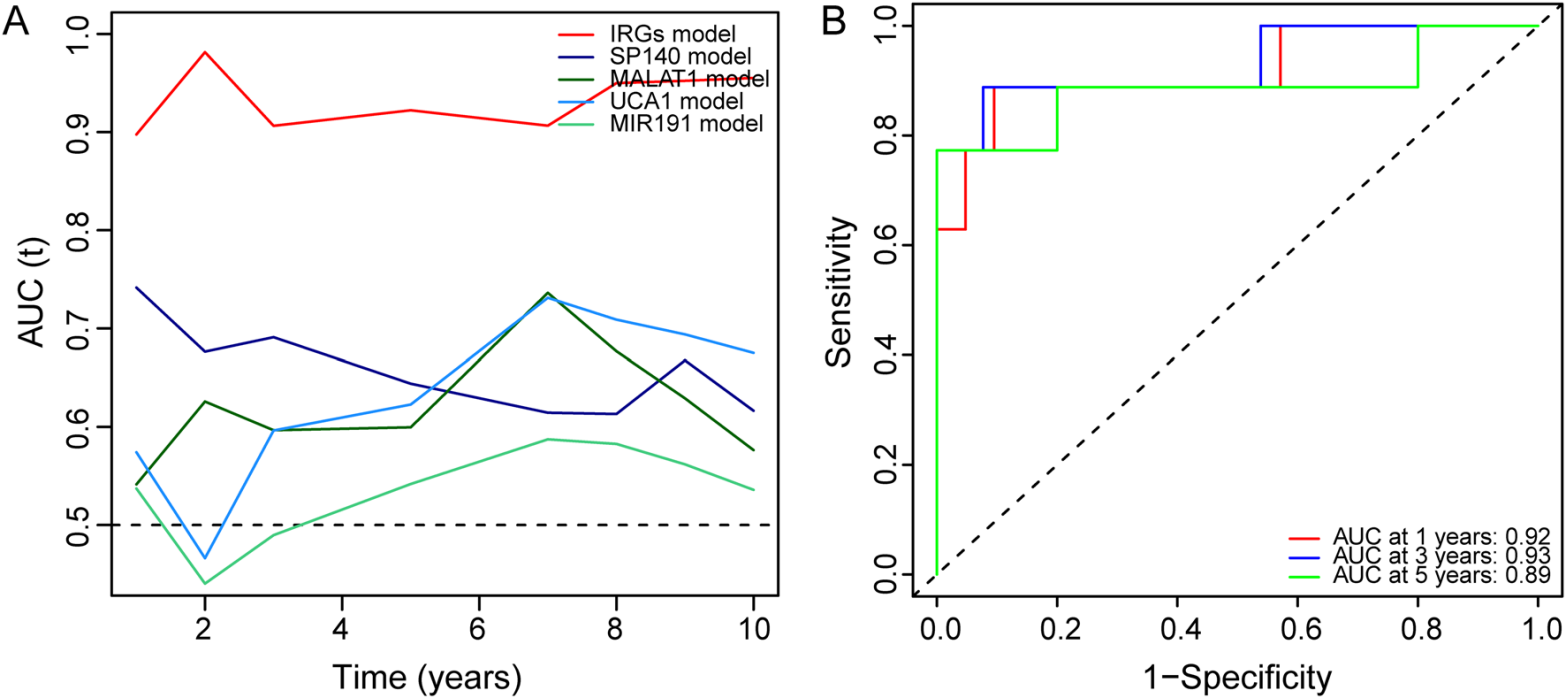
Comparison of IRGs signature with other prognostic biomarkers and verification in an independent cohort. (**A**) Time-dependent ROC curve of IRGs signature compared to that other prognostic biomarkers. (**B**) The ROC curve of the IRGs signature predicting survival in an independent cohort.

To determine whether the IRGs signature has a better diagnostic capacity for OS patient survival, we conducted receiver operating characteristic (ROC) analysis of the IRG signature along with other known prognostic biomarkers (SP140, MALAT1, UCA1, and MIR191) in the training cohort. The results showed that the area under the curve (AUC) of the IRGs signature was increased compared to that for other known biomarkers (Fig. 5A), indicating that the IRG signature was a better prognostic biomarker and provided better stability and reliability in predicting the survival of OS patients. To further examine the prognostic value of the IRG signature, we conducted the ROC analysis in another independent cohort (GSE39055). The results showed that the AUCs were 0.92, 0.93, and 0.89 at 1, 3, and 5 years, respectively (Fig. 5B), suggesting that the IRG can also predict the survival of OS patients in other independent cohorts.

### Independence of the IRGs signature in survival prediction from clinicopathological factors

**Figure 6:**
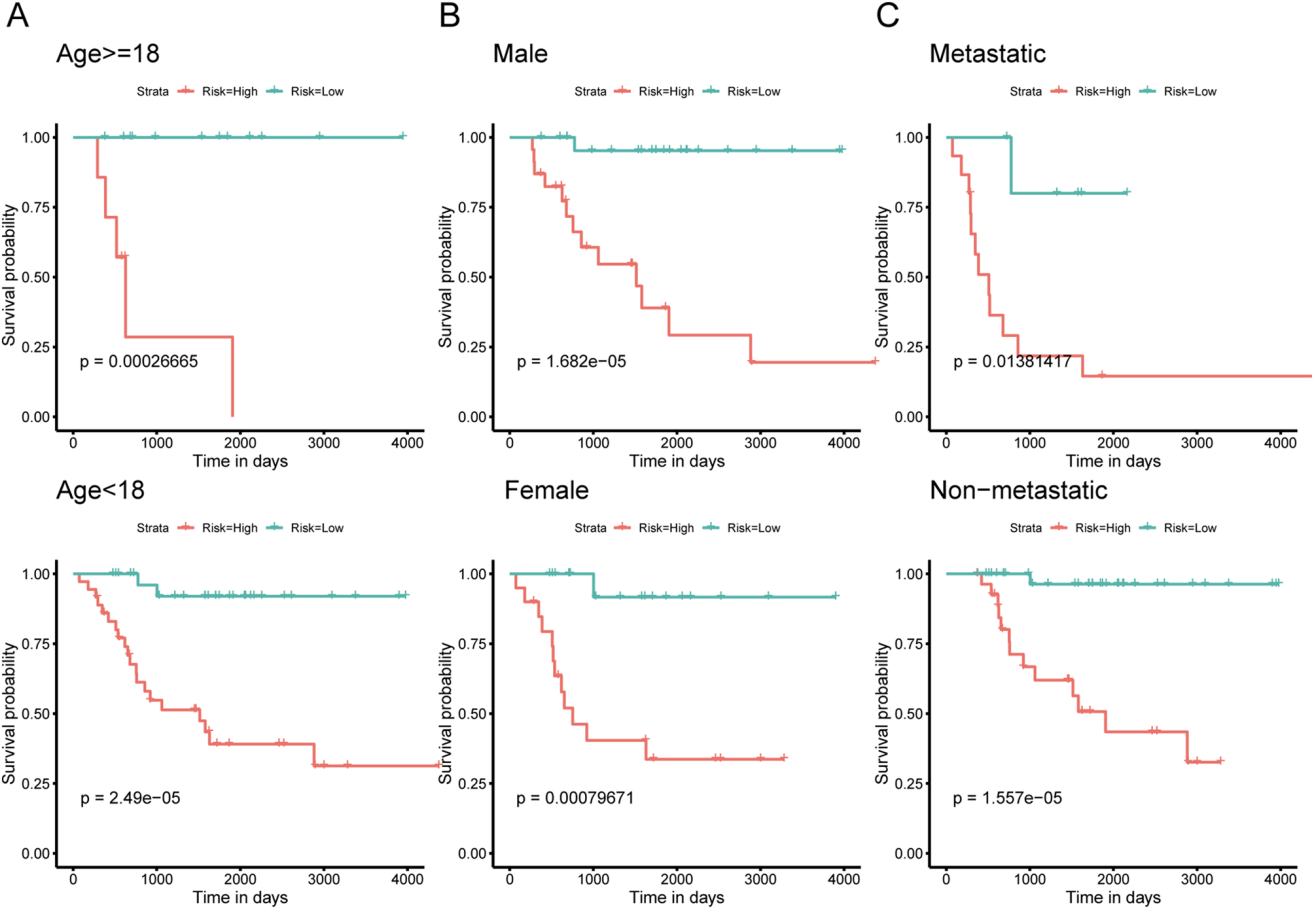
Kaplan–Meier curves of patients with OS in different clinical subgroups. (**A**) Kaplan–Meier curve for OS patients aged < 18 years and those aged ≥ 18 years. (**B**) Kaplan–Meier curve of male and female patients with OS. (**C**) Kaplan–Meier curve of metastatic and non-metastatic OS patients.

An important feature of a good prognostic biomarker is that it should be independent of clinicopathological prognostic factors. Clinicopathological characteristics, such as the patient's age, sex, and metastasis, are also considered to be the main factors that determine the prognosis of OS patients. To evaluate the independence and applicability of the IRGs signature, we regrouped patients according to different clinicopathological characteristics and performed Kaplan–Meier survival analysis. The Kaplan–Meier curve showed that regardless of sex, age, and metastasis, the survival time of OS patients in the low-risk group was significantly prolonged (p < 0.05, Fig. 6A–C). All of results indicated that the IRGs signature showed satisfactory applicability when grouping patients according to different clinicopathological characteristics. Univariate and multivariate COX regression also suggested that the signature is an independent indicator for predicting the prognosis of OS patients (Table 3).

**Table 3:** Univariate and multivariate Cox regression models of the IRGs signature in predicting survival.

Variables	Univariate Cox		Multivariate Cox	
	HR (95% CI)	p value	HR (95% CI)	p value
Gender	0.712 (0.334–1.516)	0.377869	0.608 (0.273–1.352)	0.222178
Age	0.918 (0.347–2.423)	0.863671	2.231 (0.768–6.478)	0.140079
Metastatic	0.212 (0.099–0.454)	6.61E−05	0.182 (0.077–0.434)	0.000119
Stage	0.398 (0.120–1.326)	0.133626	0.270 (0.0727–1.004)	0.050698
Site	2.241 (1.070–4.494)	0.032372	1.669 (0.737–3.779)	0.218905
Risk score	18.088 (4.273–76.561)	8.39E−05	13.196 (3.010–57.860)	0.000624

### Relationship between the prognostic signature and clinical characteristics

**Figure 7:**
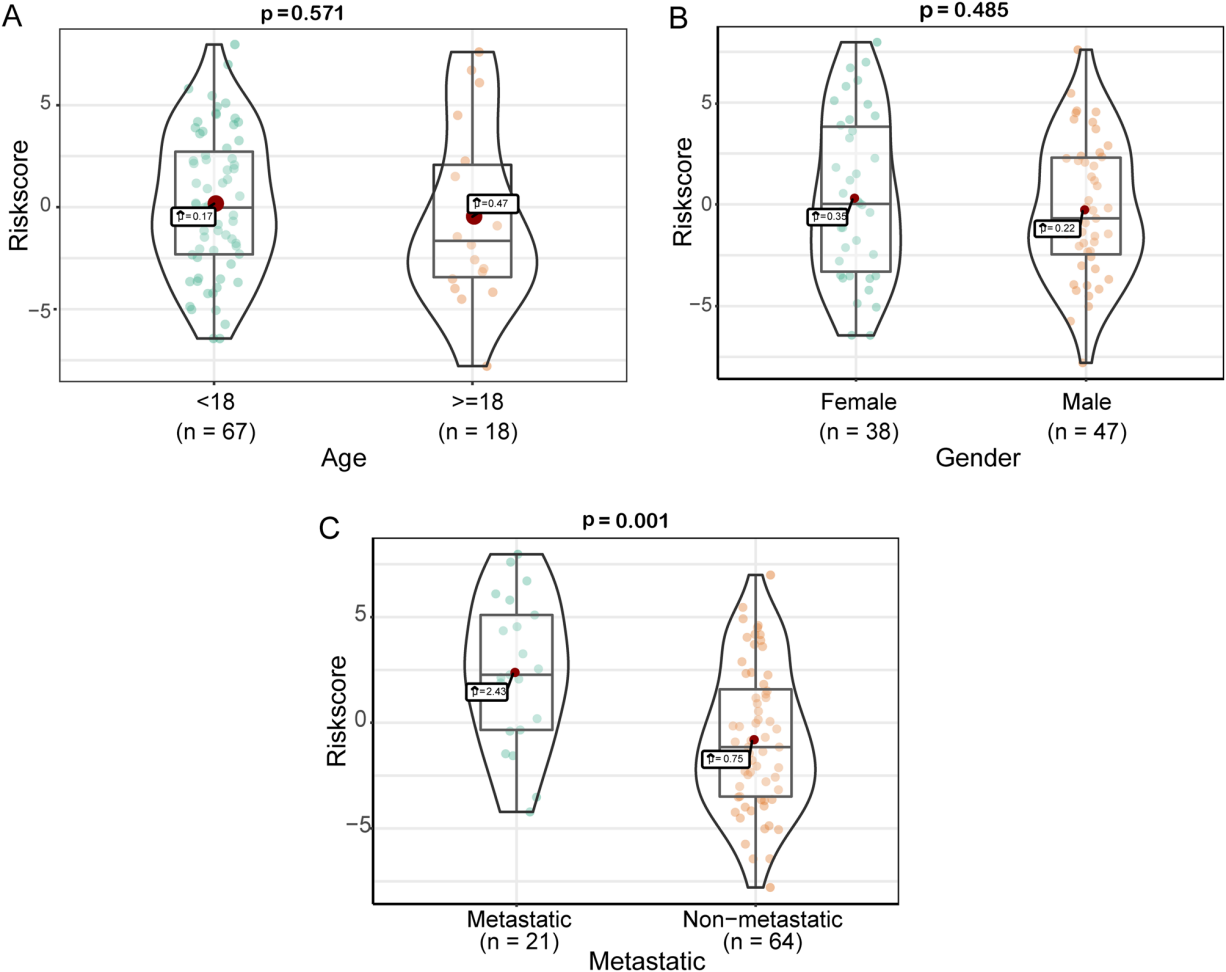
Correlation analysis between prognostic signature and clinical characteristics. (**A**) Correlation between prognosis-associated IRGs signature and age. (**B**) Correlation between prognosis-associated IRGs signature and sex. (**C**) Correlation between prognostic-associated IRGs signature and metastasis. The boxes indicate the middle 50% of the similarities; the upper and lower boundaries indicate 75% and 25%. The two ends of the violins represent the maximum and minimum values. n: number of cases of OS.

The relationship between clinical characteristics, such as metastasis, age, grade, and the risk score based on the prognosis-associated IRGs signature, was analyzed to validate the accuracy of the prognostic signature further. The results showed that metastasis groups had a significantly higher risk score than non-metastasis groups (Fig. 7C, p = 0.001). However, no significant association was observed between age (Fig. 7A, p = 0.531), sex (Fig. 7B, p = 0.485), and risk score.

## Discussion

OS is the most common bone malignancy in children and adolescents, and it is also one of the main causes of cancer-related deaths in this age group^18^. Evidence demonstrates that the immune response defines the tumor’s microenvironment. In particularly, immune cell disorders often cooccur with tumors and are considered an essential driver of OS development^19,20^. In this study, we analyzed the DEIRGs of the OS and control samples from TARGET and GTEx databases to identify new prognostic biomarkers by constructing a prognostic IRGs signature.

Related studies show that chemotaxis, adhesion of leukocyte, and innate immunity are dysfunctional in the OS microenvironment, thereby reducing the immune response to OS cells^21–23^. PI3K/AKT signaling pathway^24^, MAPK signaling pathway^25^ and JAK-STAT signaling pathway^26^ have been extensively studied in the OS. Furthermore, the activation of these signaling pathways is strongly linked to the growth and metastasis of OS cells. Although natural killer cell-mediated cytotoxicity is the host’s first-line anti-cancer defens^27^, the immune response is a seemingly double-edged sword in the OS microenvironment, as a dysregulated immune response is conducive to the occurrence and development of tumors.

In total, in our study, we obtained 604 DEIRGs. Of note, we identified 10 hub IRGs, namely CXCR3, CXCR4, CCR5, CCL5, CXCL10, CXCL12, CXCL16, OPRL1, S1PR1, and GAL. Among them, CXCR3^28^, CXCR4^29^, CCR5^30^, CCL5^31^, CXCL16^32^, CXCL10^33^, CXCL12^34^ and GAL^35^ have been widely studied in OS, and are involved in the occurrence, metastasis, and angiogenesis of OS. OPRL1 encodes proteins that are endogenous opioid-related neuropeptides and nociceptin/orphanin receptors, which plays a key role in pain perception and nociception^36,37^. The high expression of OPRL1 in OS may be related to cancerous pain. The coding product of the SIPR1 gene is a receptor protein that is similar to the G-protein-coupled receptor. When SIPR1 was combined with ligand S1P, the growth, invasion, and metastasis of lung cancer, ovarian cancer, and colon cancer are enhanced^38–40^. Hence, we can speculate that SIPR1 is pivotal in OS. Considering the similarity between molecular functions and cell components of hub IRGs, and through the ranking of semantic similarity, we discovered that CCR5, CXCL12 and CXCR4 are the most closely related genes. CCR5, CXCL12 and CXCR4 genes encode chemokine receptors or ligands, which plays a vital part role in the initiation and growth of OS^29,41,42^. These findings further support the reliability of our study.

Previous research has shown that IRGs are closely related to OS metastasis and prognosis^43^. For example, Koirala et al.^44^ found that immune cell infiltration and PD-L1 expression in the tumor microenvironment were independent risk factors for OS. Li Bo et al.^45^ reported that CXC12 acts as a driver in OS metastasis and immune response, and knocking down CXC12 could effectively inhibit OS progression. Moreover, IRGs signatures have attracted widespread attention and have been used to predict metastasis and prognosis of different tumors^46–48^. Therefore, in order to further explore the value of IRGs in OS prognosis, we constructed a prognostic signature consisting of 13 prognostic-associated DEIRGs, which has a high diagnostic prognostic efficacy. The high expression lever of GNRH1^49^, BRAF^50^, PSMD10^51^ and VEGFA^52^ closely correlated with the growth, metastasis, and angiogenesis of OS. The high expression of GAL^53,54^, TNFRSF11B^55^ and STC2^56^ are linked to prostate cancer and colorectal cancer development and a worse prognosis. The abnormally high expression of BMP8A is an independent factor for the progression and poor prognosis of thyroid carcinoma^57^. CORT is an endogenous cyclic neuropeptide that can regulate the growth and metastasis of lung cancer and thyroid cancer^58,59^, and it also regulates the inflammatory response by inhibiting the immune infiltration^60^. Granulin a (GRNA) is a 6 kDa peptide hydrolyzed from PGRN, which can effectively inhibit the growth and invasion of human hepatoma cells^61^. The high expression of VAV1 is a positive prognostic factor for early invasive breast cancer^62^. Zong et al.^63^ found that the overexpression of SDC3 can significantly inhibit the proliferation and metastasis of mesenchymal tumor cells. Wu et al.^64^ found that miR20a-5p promotes the proliferation, migration, and invasion of head and neck squamous cell carcinoma by down-regulating TNFRSF21. Another study found that TNFRSF21 also plays an important role in regulating leukocyte infiltration^65^. obviously, the results of our analysis are consistent with the results of previous studies, which further confirms that this signature has a high value for the prognosis of OS.

To date, a lot of OS prognostic molecules have been found, including MALAT1^9^, UCA1^10^ and miR191^11^. Most of these were based on single-gene prognosis studies. Existing studies have found that the occurrence and development of tumors are not caused by changes in single genes, but are the result of a series of gene changes^66^. In addition, the use of single genes cannot avoid the differences caused by individual heterogeneity. Most importantly, these studies did not use large samples to fully explore the relationship between genes and the prognosis of OS. In this study, 13 prognostic IRGs were identified by univariate cox regression and iterative LASSO cox regression analysis for the risk stratification of OS patients. Extensive analyses proved that this prognostic signature has a higher diagnostic value than pre-existing models. Recently, Shi et al.^67^ also constructed a prognostic signature that consisted of three DEGs (MYC, CPE, and LY86) in OS. However, the DEGs in their study came from metastatic and non-metastatic patients and lacked a normal control sample. Therefore, the gene included in the signature did not reflect the pathological characteristics of the occurrence and development of OS. Our signature was verified by an independent verification set, which has a high diagnostic efficiency compared to that with other biomarkers. However, our research also has some unavoidable limitations and deficiencies. First, in the study, we used normal muscle tissue as a control group. Therefore, compared with normal bone tissue, there may be a certain difference in the expression of IRGs. In addition, due to the lack of protein expression profile data for OS, we used gene expression profile data, which may not fully reflect the biological characteristics of OS. After all, protein is the executor of the function. Above all, there is still a lack of large sample data sets and clinical samples to verify the accuracy of the results of this analysis.

## Conclusion

In summary, we developed an IRGs signature that is a prognostic indicator in OS patients, and further verified it in an independent cohort. Hence, the signature might serve as potential prognostic indicator to identify outcome of OS and facilitate personalized management of the high-risk patients.

## Materials and methods

### Data processing and screening

Therapeutically Applicable Research to Generate Effective Treatments database (TARGET; https://ocg.cancer.gov/programs/target) is an open database for childhood tumors that seeks to identify molecular changes in the occurrence and progression of pediatric cancer using an integrated genomic approach to assist researchers in developing effective treatments. The Genotype-Tissue Expression (GTEx, https://www.gtexportal.org/home/) database^68^ provides transcriptome data of various normal human tissues. Gene Expression Omnibus database (GEO, https://www.ncbi.nlm.nih.gov/geo/) is a gene expression database created and maintained by NCBI, which contains high-throughput gene expression data and gene chip expression data submitted by research institutions around the world. We downloaded the gene expression profiles and the corresponding clinical data of OS from the TARGET database, including 88 OS samples, and obtained the normal muscle tissue gene expression profile data set from the GTEx database as a control group, including 396 muscle tissue samples. Then we applied the R software (Version 3.3.3, https://www.r-project.org/) sva package^69^ to merge the raw data (CEL files) of the two sets. Subsequently, we used the Limma package^70^ to screen DEGs between the OS tissue and normal muscle tissue. The cut-off value was | log_2_ fold change (log_2_FC) |> 1 and adj. p < 0.05. We downloaded and organized the IRGs list from the ImmPort (https://immport.niaid.niaid.gov) database, selected DEIRGs from DEGs and used them for our analysis.

### Functional correlation analysis of DEIRGs

GO is a tool for annotating genes and their products, which aid the integration and utilization of biological data^71^. KEGG is a database integrating genomics, chemistry, and system function information, which provides currently known biological metabolic signaling pathways^72^. The clusterProfiler package^73^ was used to perform GO and KEGG enrichment analysis on DEIRGs; p < 0.05 was used as a cut-off value for significant gene enrichment. The Search Tool for the Retrieval of Interacting Genes online tool (STRING, https://www.string-db.org/, Version: 11.0)^74^ and Cytoscape software^75^ were used to construct the PPI network for DEIRGs, and the hub IRGs were screened using the cytoHubba plug-in^76^. The hub IRGs selection criteria shortlisted the top 10 DEGs through the maximum correlation standard algorithm. Based on the semantic similarity of GO terms, GOSemSim package^77^ was used to compute closeness of the relationship between the molecular function and cell localization among 10 hub IRGs, and used the average functional similarity to rank the 10 hub IRGs^78^. The results were visualized by the ggplot2 package^79^.

### Identification and assessment of the prognostic signature

To develop the optimal signature for predicting OS prognosis based on IRGs, we performed univariate Cox regression analysis on the obtained DEIRGs, and selected IRGs related to prognosis with a screening criterion of p < 0.05. Next, we used the glmnet (https://CRAN.R-project.org/package=glmnet) package^80^ to perform a machine learning algorithm-iterative LASSO Cox regression analysis on prognostic-associated IRGs to construct the optimal prognosis signature. LASSO is highly dependent on seeds and requires cross-validation to select samples randomly. Once the seeds are replaced, the optimal lambda and resulting features change. Iterative LASSO regression was used to select high-frequency features, such as consensus genes, according to the frequency sequence of features after several runs of LASSO. Then, the consensus genes were sequentially included in the Cox model. After the AUC of ROC reached a peak, the genes were not included. At this point, the model is optimal and contains the least features^81^. We counted the consensus genes for which the frequency exceeded 50 after 500 LASSO regressions. Then we fit the expression levels of the consensus genes into a variable through the iterative LASSO cox regression to construct the optimal prognosis signature of OS. Next, we scored each sample with the optimal signature and divided the patients into a high- or low-risk group, according to the median of the score. Finally, we used R software to draw a risk factor association chart to display the survival status.

### Comparison of signature with other known prognostic biomarkers and verification in an independent cohort

Many prognostic markers for patients with OS have been previously determined. SP140 has been identified as a promising prognostic marker for OS patients^8^, and the expression of MALAT1 has been shown to be associated with a worse prognosis for OS patients^9^. UCA1 expression may be an independent prognostic indicator for predicting a poor prognosis in patients with OS^10^. In addition, miR191 is highly expressed in the serum of patients with osteosarcoma and is positively correlated with clinical stage^11^. In order to determine whether our signature has a better ability to predict patient survival than known biomarkers, we conducted a ROC comparative analysis of the signature and other biomarkers. Good prognostic markers should also have a high predictive prognostic performance in other independent cohorts. To test the utility of the signature in this study, we verified it with another independent cohort (GSE39055). Details of the GSE39055 dataset are shown in Supplementary Table 1.

### Subgroup survival analysis, signature clinical value evaluation

An important feature of a good prognostic marker is that it should be independent of the currently used clinicopathological prognostic factors. To evaluate the independence and applicability of this signature, we regrouped OS patients according to different clinicopathological characteristics, and then performed Kaplan–Meier survival analysis for their subgroups. We performed univariate and multivariate Cox regressions on clinicopathological characteristics and the signature to evaluate whether the signature is an important prognostic factor.

### Correlation analysis of prognostic signature and clinical characteristics

To further evaluate the correlation between the risk score based on the prognosis-associated IRGs signature and clinical characteristics, we classified patients according to age, sex, and distant metastatic status. Then we used the ggstatsplot (https://github.com/IndrajeetPatil/ggstatsplot) package to analyze the correlation between the risk score and the aforementioned. The results are shown in the ggplot2 package.

## Supplementary information

Supplementary Information.

## References

[1] Bielack SS (2002). Prognostic factors in high-grade osteosarcoma of the extremities or trunk: An analysis of 1,702 patients treated on neoadjuvant cooperative osteosarcoma study group protocols. J. Clin. Oncol..

[2] Liu, K. *et al.* The Sp1/FOXC1/HOTTIP/LATS2/YAP/β-catenin cascade promotes malignant and metastatic progression of osteosarcoma. Mol. Oncol**.** Advance online publication; 10.1002/1878-0261.12760 (2020).

[3] Wang Y, Huang Y, Xiang P, Tian W (2017). LncRNA expression and implication in osteosarcoma: A systematic review and meta-analysis. Onco Targets Ther..

[4] Chen L (2020). Mild microwave ablation combined with HSP90 and TGF-β1 inhibitors enhances the therapeutic effect on osteosarcoma. Mol. Med. Rep..

[5] Shankar GM (2017). The role of revision surgery and adjuvant therapy following subtotal resection of osteosarcoma of the spine: A systematic review with meta-analysis. J. Neurosurg. Spine..

[6] Zhang M, Zhang X (2015). Association of MMP-2 expression and prognosis in osteosarcoma patients. Int. J. Clin. Exp. Pathol..

[7] Rosemann M (2014). A Rb1 promoter variant with reduced activity contributes to osteosarcoma susceptibility in irradiated mice. Mol. Cancer..

[8] Hong W (2020). Immune-related prognosis biomarkers associated with osteosarcoma microenvironment. Cancer Cell Int..

[9] Wang J, Sun G (2017). FOXO1-MALAT1-miR-26a-5p feedback loop mediates proliferation and migration in osteosarcoma cells. Oncol. Res..

[10] Wen JJ, Ma YD, Yang GS, Wang GM (2017). Analysis of circulating long non-coding RNA UCA1 as potential biomarkers for diagnosis and prognosis of osteosarcoma. Eur. Rev. Med. Pharmacol. Sci..

[11] Wang T, Ji F, Dai Z, Xie Y, Yuan D (2015). Increased expression of microRNA-191 as a potential serum biomarker for diagnosis and prognosis in human osteosarcoma. Cancer Biomark..

[12] Sanchez-Diaz PC (2014). In silico functional analyses and discovery of survival-associated microRNA signatures in pediatric osteosarcoma. Oncoscience..

[13] Tuohy JL (2020). Immune dysregulation and osteosarcoma: *Staphylococcus aureus* downregulates TGF-β and heightens the inflammatory signature in human and canine macrophages suppressed by osteosarcoma. Vet. Comp. Oncol..

[14] Gentles AJ (2015). The prognostic landscape of genes and infiltrating immune cells across human cancers. Nat. Med..

[15] Shen H (2019). Predictive biomarkers for immune checkpoint blockade and opportunities for combination therapies. Genes Dis..

[16] Topalian SL, Drake CG, Pardoll DM (2015). Immune checkpoint blockade: A common denominator approach to cancer therapy. Cancer Cell.

[17] Wu W (2020). FGD1 promotes tumor progression and regulates tumor immune response in osteosarcoma via inhibiting PTEN activity. Theranostics..

[18] Anderson ME (2016). Update on survival in osteosarcoma. Orthop. Clin. N. Am..

[19] Kim R, Emi M, Tanabe K (2007). Cancer immunoediting from immune surveillance to immune escape. Immunology.

[20] Heymann MF, Lézot F, Heymann D (2019). The contribution of immune infiltrates and the local microenvironment in the pathogenesis of osteosarcoma. Cell Immunol..

[21] Lauri D, Needham L, Martin-Padura I, Dejana E (1991). Tumor cell adhesion to endothelial cells: Endothelial leukocyte adhesion molecule-1 as an inducible adhesive receptor specific for colon carcinoma cells. J. Natl. Cancer Inst..

[22] Byrum ML, Pondenis HC, Fredrickson RL, Wycislo KL, Fan TM (2016). Downregulation of CXCR4 expression and functionality after zoledronate exposure in canine osteosarcoma. J. Vet. Intern. Med..

[23] Piazzi M (2020). Expression of the double-stranded RNA-dependent kinase PKR influences osteosarcoma attachment independent growth, migration, and invasion. J. Cell Physiol..

[24] Zhao GS (2018). TSSC3 promotes autophagy via inactivating the Src-mediated PI3K/Akt/mTOR pathway to suppress tumorigenesis and metastasis in osteosarcoma, and predicts a favorable prognosis. J. Exp. Clin. Cancer Res..

[25] Zhu J (2017). Escin induces caspase-dependent apoptosis and autophagy through the ROS/p38 MAPK signalling pathway in human osteosarcoma cells in vitro and in vivo. Cell Death Dis..

[26] Liu Y, Guan J, Chen X (2019). Identification of differentially expressed genes under the regulation of transcription factors in osteosarcoma. Pathol. Oncol. Res..

[27] Kubista B, Trieb K, Blahovec H, Kotz R, Micksche M (2002). Hyperthermia increases the susceptibility of chondro- and osteosarcoma cells to natural killer cell-mediated lysis. Anticancer Res..

[28] Tang Y, Gu Z, Fu Y, Wang J (2019). CXCR3 from chemokine receptor family correlates with immune infiltration and predicts poor survival in osteosarcoma. Biosci. Rep..

[29] Jiang K (2019). SDF-1/CXCR4 axis facilitates myeloid-derived suppressor cells accumulation in osteosarcoma microenvironment and blunts the response to anti-PD-1 therapy. Int. Immunopharmacol..

[30] Wang SW (2015). CCL5/CCR5 axis induces vascular endothelial growth factor-mediated tumor angiogenesis in human osteosarcoma microenvironment. Carcinogenesis.

[31] Sun K (2017). High CCL5 expression is associated with osteosarcoma metastasis and poor prognosis of patients with osteosarcoma. Mol. Med. Rep..

[32] Zhang F, Huang W, Sheng M, Liu T (2015). MiR-451 inhibits cell growth and invasion by targeting CXCL16 and is associated with prognosis of osteosarcoma patients. Tumour Biol..

[33] Flores RJ (2017). A novel prognostic model for osteosarcoma using circulating CXCL10 and FLT3LG. Cancer.

[34] Pollino S (2019). CXCR4 in human osteosarcoma malignant progression. The response of osteosarcoma cell lines to the fully human CXCR4 antibody MDX1338. J. Bone Oncol..

[35] Miao JH (2014). Knockdown of galectin-1 suppresses the growth and invasion of osteosarcoma cells through inhibition of the MAPK/ERK pathway. Oncol. Rep..

[36] Toll L, Bruchas MR, Calo' G, Cox BM, Zaveri NT (2016). Nociceptin/orphanin FQ receptor structure, signaling, ligands, functions, and interactions with opioid systems. Pharmacol. Rev..

[37] Kiguchi N, Ding H, Ko MC (2016). Central N/OFQ-NOP Receptor System in Pain Modulation. Adv Pharmacol..

[38] Rostami N (2019). S1PR1 as a novel promising therapeutic target in cancer therapy. Mol. Diagn. Ther..

[39] Nagahashi M (2014). Sphingosine-1-phosphate in chronic intestinal inflammation and cancer. Adv. Biol. Regul..

[40] Dai L (2017). Sphingosine kinase 1/sphingosine-1-phosphate (S1P)/S1P receptor axis is involved in ovarian cancer angiogenesis. Oncotarget..

[41] Wang SW (2012). CCL5 and CCR5 interaction promotes cell motility in human osteosarcoma. PLoS ONE.

[42] Neklyudova O (2016). Altered CXCL12 expression reveals a dual role of CXCR4 in osteosarcoma primary tumor growth and metastasis. J. Cancer Res. Clin. Oncol..

[43] Miwa S (2019). Therapeutic targets for bone and soft-tissue sarcomas. Int. J. Mol. Sci..

[44] Koirala P (2016). Immune infiltration and PD-L1 expression in the tumor microenvironment are prognostic in osteosarcoma. Sci. Rep..

[45] Li B (2018). Epigenetic regulation of CXCL12 plays a critical role in mediating tumor progression and the immune response in osteosarcoma. Cancer Res..

[46] Wu J, Zhao Y, Zhang J, Wu Q, Wang W (2019). Development and validation of an immune-related gene pairs signature in colorectal cancer. Oncoimmunology..

[47] Lin P (2019). Development of a prognostic index based on an immunogenomic landscape analysis of papillary thyroid cancer. Aging.

[48] Tan W (2020). Construction of an immune-related genes nomogram for the preoperative prediction of axillary lymph node metastasis in triple-negative breast cancer. Artif. Cells Nanomed. Biotechnol..

[49] von Alten J (2006). GnRH analogs reduce invasiveness of human breast cancer cells. Breast Cancer Res. Treat..

[50] Kim B (2014). Clinical meaning of BRAF mutation in Korean patients with advanced colorectal cancer. World J. Gastroenterol..

[51] Cheng L (2020). Gankyrin promotes osteosarcoma tumorigenesis by forming a positive feedback loop with YAP. Cell Signal..

[52] Wu H, Zhang J, Dai R, Xu J, Feng H (2019). Transferrin receptor-1 and VEGF are prognostic factors for osteosarcoma. J. Orthop. Surg. Res..

[53] Shih TC (2018). Targeting galectin-1 impairs castration-resistant prostate cancer progression and invasion. Clin. Cancer Res..

[54] Nagayoshi K (2015). Galanin plays an important role in cancer invasiveness and is associated with poor prognosis in stage II colorectal cancer. Oncol. Rep..

[55] Marzioni D (2009). Expression of basic fibroblast growth factor, its receptors and syndecans in bladder cancer. Int. J. Immunopathol. Pharmacol..

[56] Ieta K (2009). Clinicopathological significance of stanniocalcin 2 gene expression in colorectal cancer. Int. J. Cancer..

[57] Liu K, Gao M, Qin D, Wang H, Lu Q (2019). Serous BMP8A has clinical significance in ultrasonic diagnosis of thyroid cancer and promotes thyroid cancer cell progression. Endocr. Metab. Immune Disord. Drug Targets..

[58] Cassoni P (2006). Ghrelin and cortistatin in lung cancer: Expression of peptides and related receptors in human primary tumors and in vitro effect on the H345 small cell carcinoma cell line. J. Endocrinol. Investig..

[59] Cassoni P (2002). Cortistatin-14 inhibits cell proliferation of human thyroid carcinoma cell lines of both follicular and parafollicular origin. J. Endocrinol. Investig..

[60] Delgado-Maroto V (2017). The neuropeptide cortistatin attenuates experimental autoimmune myocarditis via inhibition of cardiomyogenic T cell-driven inflammatory responses. Br. J. Pharmacol..

[61] Chen X (2017). Interaction between granulin A and enolase 1 attenuates the migration and invasion of human hepatoma cells. Oncotarget..

[62] Grassilli S (2014). High nuclear level of Vav1 is a positive prognostic factor in early invasive breast tumors: A role in modulating genes related to the efficiency of metastatic process. Oncotarget..

[63] Zong F (2010). Effect of syndecan-1 overexpression on mesenchymal tumour cell proliferation with focus on different functional domains. Cell Prolif..

[64] Wu H (2018). Upregulated miR20a5p expression promotes proliferation and invasion of head and neck squamous cell carcinoma cells by targeting of TNFRSF21. Oncol. Rep..

[65] Schmidt CS (2005). Resistance to myelin oligodendrocyte glycoprotein-induced experimental autoimmune encephalomyelitis by death receptor 6-deficient mice. J. Immunol..

[66] Matin A, Nadeau JH (2001). Sensitized polygenic trait analysis. Trends Genet..

[67] Shi Y (2020). A risk signature-based on metastasis-associated genes to predict survival of patients with osteosarcoma. J. Cell Biochem..

[68] multitissue gene regulation in humans (2015). Human genomics. the genotype-tissue expression (GTEx) pilot analysis. Science.

[69] Parker HS (2014). Preserving biological heterogeneity with a permuted surrogate variable analysis for genomics batch correction. Bioinformatics.

[70] Ritchie ME (2015). limma powers differential expression analyses for RNA-sequencing and microarray studies. Nucleic Acids Res..

[71] Yu G, Wang LG, Han Y, He QY (2012). clusterProfiler: An R package for comparing biological themes among gene clusters. OMICS..

[72] Harris MA (2004). The Gene Ontology (GO) database and informatics resource. Nucleic Acids Res..

[73] Kanehisa M, Furumichi M, Tanabe M, Sato Y, Morishima K (2017). KEGG: New perspectives on genomes, pathways, diseases and drugs. Nucleic Acids Res..

[74] Szklarczyk D (2019). STRING v11: Protein-protein association networks with increased coverage, supporting functional discovery in genome-wide experimental datasets. Nucleic Acids Res..

[75] Smoot ME, Ono K, Ruscheinski J, Wang PL, Ideker T (2011). Cytoscape 2.8: New features for data integration and network visualization. Bioinformatics.

[76] Chin CH (2014). cytoHubba: Identifying hub objects and sub-networks from complex interactome. BMC Syst. Biol..

[77] Yu G (2010). GOSemSim: An R package for measuring semantic similarity among GO terms and gene products. Bioinformatics.

[78] Sevilla JL (2005). Correlation between gene expression and GO semantic similarity. IEEE/ACM Trans. Comput. Biol. Bioinf..

[79] Ito K, Murphy D (2013). Application of ggplot2 to pharmacometric graphics. CPT Pharmacometrics Syst. Pharmacol..

[80] Engebretsen S, Bohlin J (2019). Statistical predictions with glmnet. Clin. Epigenetics..

[81] Tibshirani R (1997). The lasso method for variable selection in the Cox model. Stat. Med..

