# Supplementary material for: Development of a novel immune-related genes prognostic signature for osteosarcoma
Source: Sci Rep. 2020 Oct 27;10:18402. doi: 10.1038/s41598-020-75573-w (PMC7591524; doi:10.1038/s41598-020-75573-w)
Supplement: Supplementary file 1 — Supplementary Information. [file 41598_2020_75573_MOESM1_ESM.docx]

**Development of a novel immune-related genes prognostic signature for osteosarcoma**

Zuo-long Wu #, Ya-jun Deng #, Guang-zhi Zhang #, En-hui Ren, Wen-hua Yuan; Qi-qi Xie*

| Sample | age | gender | necrosis | recurrence | OS. times | OS |
| --- | --- | --- | --- | --- | --- | --- |

Supplementary table 1: Information on patients with osteosarcoma from GSE39055 Set.

| GSM954790 | 4 years | male | 60% necrosis | recurrence | 126.3 | dead |
| --- | --- | --- | --- | --- | --- | --- |
| GSM954791 | 11 years | male | >90% necrosis | recurrence | 19.5 | alive |
| GSM954792 | 4 years | female | 94% necrosis | non-recurrence | 82.6 | alive |
| GSM954793 | 5 years | male | >99% necrosis | non-recurrence | 61.1 | alive |
| GSM954794 | 13 years | male | 90-95% necrosis | recurrence | 30.4 | alive |
| GSM954795 | 12 years | female | 98% necrosis | non-recurrence | 200.9 | alive |
| GSM954796 | 16 years | male | 60-70% necrosis | non-recurrence | 28.2 | alive |
| GSM954797 | 12 years | male | 50% necrosis | recurrence | 151 | dead |
| GSM954798 | 10 years | female | 10-20% necrosis | non-recurrence | 34.4 | alive |
| GSM954799 | 7 years | female | 70% necrosis | recurrence | 9.4 | alive |
| GSM954800 | 16 years | female | 80% necrosis | non-recurrence | 50 | alive |
| GSM954801 | 13 years | male | 70% necrosis | recurrence | 12.9 | dead |
| GSM954802 | 12 years | male | 20% necrosis | non-recurrence | 196.1 | alive |
| GSM954803 | 11 years | female | 40% necrosis | recurrence | 33.5 | alive |
| GSM954804 | 9 years | female | 30% necrosis | non-recurrence | 124.3 | alive |
| GSM954805 | 13 years | female | >99% necrosis | non-recurrence | 65.7 | alive |
| GSM954806 | 9 years | female | 40% necrosis | recurrence | 59.2 | alive |
| GSM954807 | 15 years | female | 60-70% necrosis | non-recurrence | 50.4 | alive |
| GSM954808 | 4 years | female | >95% necrosis | non-recurrence | 74.6 | alive |
| GSM954809 | 23 years | female | >80% necrosis | recurrence | 64.6 | alive |
| GSM954810 | 8 years | male | >95% necrosis | non-recurrence | 70.9 | alive |
| GSM954811 | 11 years | female | 20-25% necrosis | recurrence | 29.9 | dead |
| GSM954812 | 16 years | male | >95% necrosis | non-recurrence | 55.6 | alive |
| GSM954813 | 5 years | male | >99% necrosis | non-recurrence | 72.1 | alive |
| GSM954814 | 13 years | male | >90% necrosis | non-recurrence | 14.2 | alive |
| GSM954815 | 9 years | female | 98% necrosis | non-recurrence | 71.9 | alive |
| GSM954816 | 10 years | male | >95% necrosis | recurrence | 24.1 | dead |
| GSM954817 | 7 years | female | 50% necrosis | recurrence | 8.9 | dead |
| GSM954818 | 11 years | male | 20% necrosis | recurrence | 14.1 | dead |
| GSM954819 | 9 years | female | 50% necrosis | recurrence | 14 | dead |
| GSM954820 | 13 years | male | 20% necrosis | recurrence | 28 | alive |
| GSM954821 | 13 years | male | 50% necrosis | non-recurrence | 0 | alive |
| GSM954822 | 17 years | male | 75-80% necrosis | recurrence | 13.5 | alive |
| GSM954823 | 9 years | male | 40% necrosis | non-recurrence | 6.1 | alive |
| GSM954824 | 33 years | female | 5% necrosis | recurrence | 25 | dead |
| GSM954825 | 25 years | male | 20% necrosis | recurrence | 2.7 | dead |
| GSM954826 | 71 years | male | 50% necrosis | non-recurrence | 32 | alive |

OS: overall survival.
